# Alterations of the Gut Microbiome Associated With the Treatment of Hyperuricaemia in Male Rats

**DOI:** 10.3389/fmicb.2018.02233

**Published:** 2018-09-19

**Authors:** Yiran Yu, Qiuping Liu, Haichang Li, Chengping Wen, Zhixing He

**Affiliations:** Institute of Basic Research in Clinical Medicine, College of Basic Medical Science, Zhejiang Chinese Medical University, Hangzhou, China

**Keywords:** hyperuricaemia, gut microbiome, allopurinol, benzbromarone, Illumina MiSeq

## Abstract

Hyperuricaemia is an important risk factor for many diseases including gout, hypertension, and type II diabetes. The gut microbiota is associated with hyperuricaemia and has also been demonstrated to play significant roles in the effects of drug therapy. This study used Illumina MiSeq sequencing to explore alterations of the gut microbiome associated with allopurinol and benzbromarone treatment in the male rat with hyperuricaemia. After drug treatment, both allopurinol and benzbromarone caused an increase of the genera *Bifidobacterium* and *Collinsella* and a decrease of the genera *Adlercreutzia* and *Anaerostipes*. In addition, allopurinol and benzbromarone caused respective unique changes in genera. The genera *Bilophila*, *Morganella*, and *Desulfovibrio* specifically decreased due to allopurinol treatment. Decreased *Butyricimonas* and *Ruminococcus* and increased *Proteus* were caused by benzbromarone treatment. The PICRUST analysis indicated that allopurinol renovated the disorder of nucleotide metabolism and benzbromarone renovated the disorder of lipid metabolism in the gut microbiota of male rats with hyperuricaemia. These findings demonstrated that the gut microbiota may be altered by the treatment of hyperuricaemia with allopurinol and benzbromarone in male rats. Such alterations of the gut microbiota could be considered as indicators of the effectiveness of drug therapy.

## Introduction

Hyperuricaemia is characterized by the elevation of blood uric acid (UA) caused by disordered purine metabolism. Hyperuricaemia is considered to be a metabolic problem, and its associations with many diseases have been observed for decades. These diseases include gout (Bardin and Richette, 2014), cardiovascular disease (Yang et al., 2012; Li et al., 2014), chronic renal disease (Talaat and el-Sheikh, 2007), and type 2 diabetes (Mantovani et al., 2016). The prevalence of hyperuricaemia has been increasing worldwide, and urate-lowering therapy has been widely used to control hyperuricaemia (Smith and March, 2015; Li et al., 2016). Numerous anti-hyperuricaemia drugs have been developed in recent years.

Allopurinol and benzbromarone are two common urate-reducing drugs with different mechanisms of action. Allopurinol is an effective xanthine oxidase inhibitor that blocks UA production (Kim et al., 2015), while benzbromarone is a uricosuric agent that promotes UA excretion (Kunishima et al., 2007). It is well known that the gut microbiome could be partially responsible for converting xanthine into UA (Barsoum and El-Khatib, 2017) and excreting 1/3 of the UA into intestinal tract (Sorensen and Levinson, 1975). Therefore, the gut microbiome is likely altered in response to alterations in UA metabolism.

Additionally, alterations in the gut microbiota are strongly associated with hyperuricaemia. The gut microbiota and its metabolites play significant roles in the pathogenesis of hyperuricaemia-related diseases, such as gout (Guo et al., 2016; Shao et al., 2017), hypertension (Jose and Raj, 2015; Karbach et al., 2016), and type 2 diabetes (Hartstra et al., 2015; Mardinoglu et al., 2016). With the development of research examining the gut microbiome, increasing evidence has demonstrated that the gut microbiota is associated not only with the pathogenesis of disease, but also with the effects of disease treatment (Rooks et al., 2014; Ananthakrishnan et al., 2017). The reason for this association is that the gut microbiome is the target of drugs and participates in the immunity and metabolism of the host (Yang et al., 2017). Therefore, studying the alterations of the gut microbiome in response to drug therapy will be helpful in choosing appropriate drugs.

To explore the alterations of the gut microbiota in hyperuricaemia treatment using allopurinol and benzbromarone, Illumina MiSeq sequencing of the 16S rRNA gene was employed to study gut microbiota profiles in the male rats with hyperuricaemia. The overall goal of this study was to measure the effects of allopurinol and benzbromarone on the gut microbiota in the hyperuricaemia model rats.

## Materials and Methods

### Hyperuricaemia Model Establishment and Drug Treatment

Twenty-four male Sprague Dawley (SD) rats weighing 200 ± 20 g (6-month-old), provided by Shanghai SLAC Laboratory Animal Co., Ltd., were randomly divided into four groups (six per group): the blank control group (Control), the hyperuricaemia model group (Model), the allopurinol treated group (Allopurinol) and the benzbromarone treated group (Benzbromarone). Rats were singly housed under 12 h/12 h light/dark cycle, constant temperature (25 ± 1°C) and constant humidity (50 ± 5%) conditions with food and water available *ad libitum*. After 7 days of adaptive feeding, three groups were fed a hyperuricaemia-inducing diet of high-fat feed containing 10% yeast extract, while the blank control group was given the normal diet.

The administration of allopurinol and benzbromarone was initiated on the third week and continued for another 6 weeks. The model and drug groups were fed with high-fat feed containing 10% yeast extract in the whole process due to expressed urate oxidase in SD rats. SD rats in the drug groups were treated with 9 mg/kg of allopurinol or benzbromarone per day, and the control and model groups were given distilled water accordingly. The dosages of drugs were calculated based on the weight of the SD rats, which was measured every 3 days.

### Sample Collection and Storage

Blood and stool samples were collected 24 h after the last drug administration. Blood was collected from the eye socket vein in each rat and centrifuged at 1300 × *g* for 10 min at 4°C after 2 h incubation. The serum was isolated and stored at −80°C for detection of serum UA, creatinine (Cr), glutamic oxaloacetic transaminase (AST) and cholesterol (CHO1). The above serum levels were measured via an enzymatic-colourimetric method, using standard test kits on a TBA-40FR automated biochemical analyzer (Toshiba Medical Sys-tems Co., Ltd., Tokyo, Japan).

Fresh stool samples were collected by stimulating SD rats excrement. Next, total DNA was extracted from stool samples using the QIAamp^®^ DNA Stool Mini Kit (Qiagen, Hilden, Germany) according to the manufacturer’s protocols. Total DNA was determined by agarose gel electrophoresis (1% w/v agarose) and quantified using a NanoDrop 2000 spectrophotometer (Thermo Scientific). DNA was stored at −80°C for Illumina MiSeq sequencing analysis.

### 16S rRNA Gene Amplification and Sequencing

For amplification of the bacterial 16S rRNA gene, specific primers (319f: 5′-AC TCCTACGGGAGGCAGCAG-3′ and 806r: 5′-GGACTACHVGGGTWTCTAAT-3′) were used to target the hypervariable region V3–V4 of the 16S rRNA gene. PCR amplification was performed in a 30 μl mixture containing 0.5 μl of DMSO, 1.0 μl of forward primer (10 mM), 1.0 μl of reverse primer (10 mM), 5.0 μl of the DNA sample, 7.5 μl of ddH_2_O and 15.0 μl of Phusion High-Fidelity PCR Master Mix with HF Buffer (NEB). The reactions were hot-started at 98°C for 30 s, followed by 30 cycles of 98°C for 15 s, 58°C for 15 s, and 72°C for 15 s, with a final extension step at 72°C for 1 min. Subsequently, the amplicons were purified according to standard procedures, quantified, pooled and sequenced with the MiSeq Reagent Kits v3 (600 cycles, Illumina) according to the manufacturer’s instructions with 20% OhiX (Illumina). The sequencing reaction was conducted by Hangzhou Guhe Information and Technology Co., Ltd., Zhejiang, China.

### Data Analysis and Bioinformatics

After sequencing, the generated FASTQ data were prepared for analysis using Quantitative Insights into Microbial Ecology (QIIME, version 1.9) (Caporaso et al., 2010). Clean reads were extracted from the raw paired end reads under the following criteria: (i) reads were truncated at any site receiving an average quality score of <20 bp over a 50-bp sliding window, and truncated reads shorter than 50 bp were discarded; (ii) exact barcode matching, two nucleotide mismatch in primer matching, and reads containing ambiguous characters were removed; (iii) only sequences that overlapped for more than 20 bp were merged according to their overlapping sequences, reads that could not be merged were discarded.

Clean reads were clustered into 16S rRNA operational taxonomic units (OTUs) with a 97% similarity cutoff using UCLUST (Edgar, 2010). Taxonomic assignment was performed using the SILVA database (Quast et al., 2013), and where necessary sequences were blasted in the NCBI database for further classification (Agarwala et al., 2016). The OTUs comprising less than 0.005% of the total number of reads, present in one sample, were filtered out. The statistical comparisons of alpha-diversity metrics were performed using the R programme package “Vegan.” Calculated beta-diversity metrics (Bray Curtis, unweighted and weighted UniFrac) were compared using the non-parametric ANOSIM measure. Principal coordinates analysis based on the beta-diversity metrics were conducted using the R package. The specific characterization of the gut microbiota was analyzed using the linear discriminant analysis (LDA) effect size (LEfSe) method^1^ (Segata et al., 2011). LEfSe uses the non-parametric Kruskal–Wallis and pair Wilcoxon rank sum tests to determine the features with significantly different abundances among the treated groups and uses LDA to assess the effect size of each feature. The LEfSe’s alpha parameter for tests was set to 0.05, and the threshold on the logarithmic score of LDA analysis was set to 2.0. Additionally, metabolic function of gut microbiota was inferred using the PICRUST that predicted the molecular functions of each sample based on 16S rRNA maker gene sequences (Langille et al., 2013). These predictions were pre-calculated for genes in KEGG database. To reveal the different predictive functions, Welch’s *t*-tests were used for two group comparisons in STAMP software (Parks et al., 2014). The significantly different functions between two groups were obtained after filtering with *p*-value <0.05.

Multiple group differences were analyzed using the Mann–Whitney non-parametric test in SPSS software 16.0. Following statistical analyses with multiple comparisons, *p* values were adjusted using the Benjamini–Hochberg method to control the false discovery rate (FDR). An adjusted *p* value of 0.05 was used as a statistically significant cutoff.

## Results

### Changes in Serum Indices in SD Rats

**FIGURE 1:**
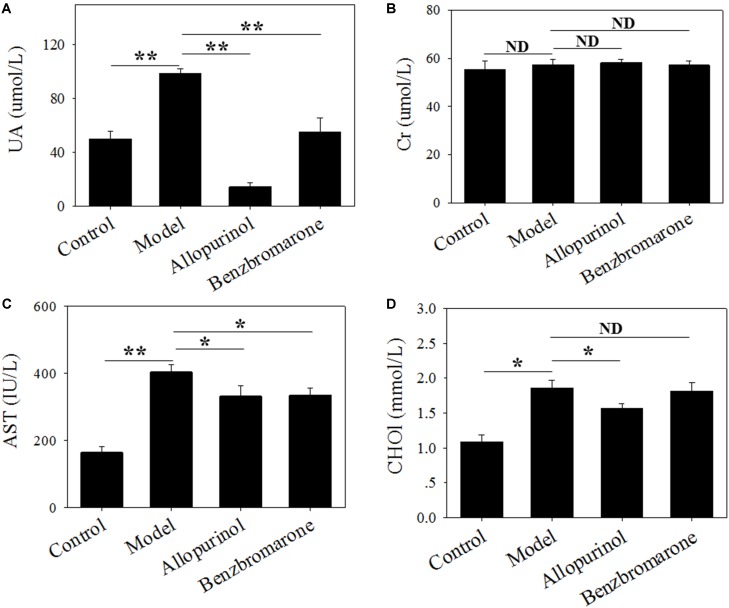
Serum uric acid **(A)**, creatinine **(B)**, glutamic oxaloacetic transaminase **(C)** and cholesterol **(D)** levels of male rats in four treatment groups. “^∗^” represents the adjusted *p* value <0.05 between two groups; “^∗∗^” represents the adjusted *p* value <0.01 between two groups; “ND” represents the adjusted *p* value >0.05 between groups.

Hyperuricaemia is characterized by a high serum urate concentration. **Figure 1** shows a significant increase (adjust *p* value <0.01) in the UA level of the model group compared to the control group, indicating that the high-fat feed successfully induced a hyperuricaemia model. Additionally, the high-fat feed significantly enhanced the serum AST (adjust *p* value <0.01) and CHO1 (adjust *p* value <0.05) levels in the hyperuricaemia rats.

In the therapeutic regimen, both allopurinol and benzbromarone interventions caused a significant (adjust *p* value <0.01) decrease in UA levels after 6 weeks of treatment compared to the model group (**Figure 1**). Interestingly, allopurinol reduced the UA level to below a normal level, but benzbromarone reduced UA to a normal level (**Figure 1**). This difference is likely due to the different mechanisms of allopurinol and benzbromarone in reducing urate. The serum AST level was also significantly reduced (adjust *p* value <0.05) by allopurinol and benzbromarone treatment but was higher than the normal level (**Figure 1**). The serum CHO1 level was significantly decreased by allopurinol but not by benzbromarone (**Figure 1**). The serum Cr level was also monitored, but no significant alteration was observed (**Figure 1**).

### Diversity of the Gut Microbiota

**FIGURE 2:**
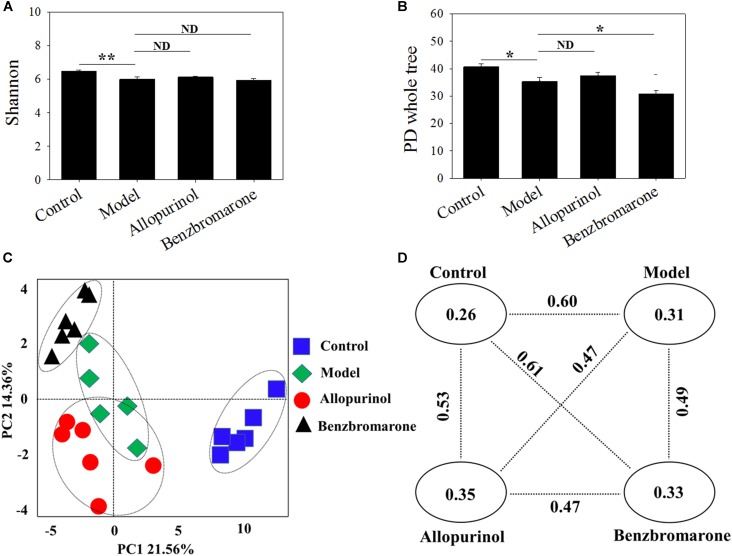
Alpha diversity indices [Shannon **(A)**; PD Whole Tree **(B)**] and Beta diversity [PCoA score plots **(C)**; unweighted UniFrac distance **(D)**] of male rats in the four groups. “^∗^” represents the adjusted *p* value <0.05 between two groups; “^∗∗^” represents the adjusted *p* value <0.01 between two groups; “ND” represents the adjusted *p* value >0.05 between groups.

The gut microbiota of the model group had a significantly lower diversity (*p* < 0.05) that that of the control group according to the Shannon and PD whole tree indices (**Figures 2A,B**). After drug treatment, there was no significant alteration in the Shannon index of the allopurinol or benzbromarone groups, but the PD whole tree was decreased in the benzbromarone group compared to the model group. The above results indicated a lower microbial richness in samples of the hyperuricaemia model, and that reducing UA did not lead to the recovery of microbial richness.

The beta-diversity among different groups using unweighted UniFrac distance was also evaluated. A scatter plot based on PCoA scores showed a clear separation of the community composition among the four groups (**Figure 2C**). PC1 and PC2 explained 21.56 and 14.36% of total variance, respectively. **Figure 2D** shows the unweighted UniFrac distance between two groups. All intra-group distance values were lower than the inter-group distance values indicating sample repeatability. The control group and benzbromarone groups has showed the greatest inter-group distance value (value = 0.61) (**Figure 2D**).

### Alterations of the Gut Microbiota

**FIGURE 3:**
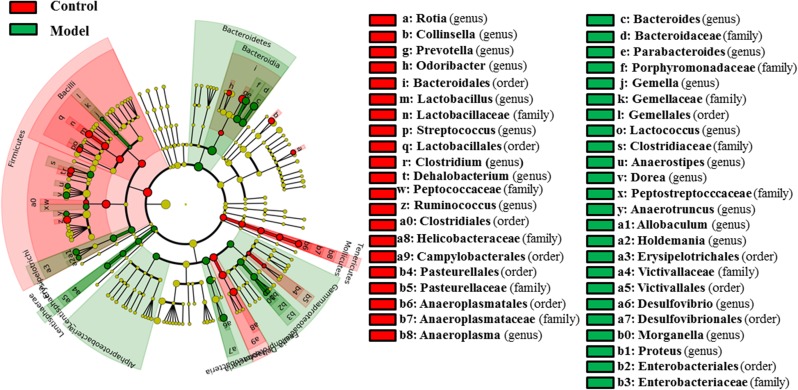
Differentially expressed taxa with the LDA scores >2.0 and adjusted *p* values <0.05 between the control rats and model rats. Differences are represented by the color of over-represented taxa: red indicating the control rats, green indicating the model rats. Circles represent phylogenetic levels from phylum (innermost circle) to genera (outermost circle).

As shown in **Figure 3**, 58 bacterial taxa were significantly distinct between the control and model groups. At the phylum level, the hyperuricaemia model rats had an increased abundance of Bacteroidetes and Lentisphaerae, and decreased abundance of Firmicutes and Tenericutes. At the genus level, 12 genera (*Bacteroides*, *Parabacteroides*, *Gemella*, *Lactococcus*, *Anaerostipes*, *Dorea*, *Anaerotruncus*, *Allobaculum*, *Holdemania*, *Desulfovibrio*, *Morganella*, and *Proteus*) were more abundant in the hyperuricaemia model rats, but another 10 genera (*Rothia*, *Collinsella*, *Prevotella*, *Odoribacter*, *Lactobacillus*, *Streptococcus*, *Clostridium*, *Dehalobacterium*, *Ruminococcus*, and *Anaeroplasma*) were less abundant in the hyperuricaemia model rats. To show the main bacterial taxa in the gut microbiome, **Supplementary Figure S1** showed the taxa with relative abundances above 1% at the phylum and genus levels. Two significantly altered phylum (Bacteroidetes and Firmicutes) and eight significantly altered genera (*Desulfovibrio*, *Bacteroides*, *Parabacteroides*, *Dorea*, *Collinsella*, *Prevotella*, *Lactobacillus*, and *Ruminococcus*) were the main significant bacterial taxa (**Supplementary Figure S1**).

**FIGURE 4:**
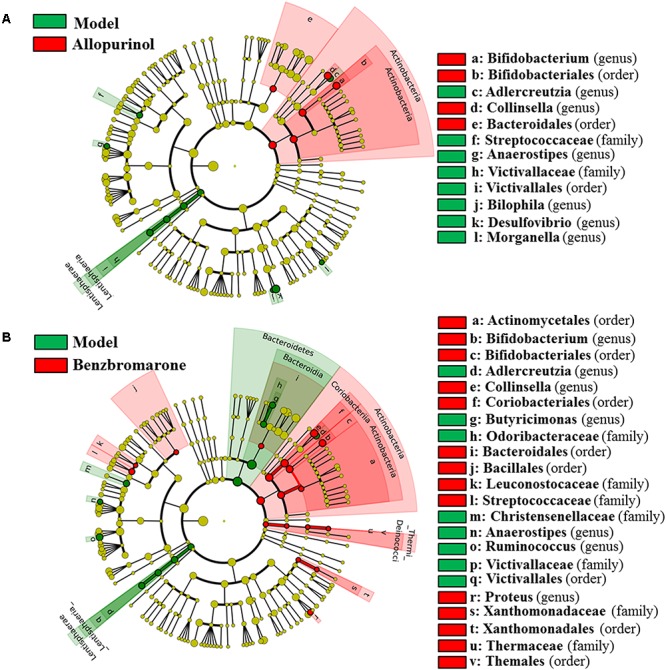
Differentially expressed taxa with the LDA scores >2.0 and adjusted *p* values <0.05 between the model rats and allopurinol-treated rats **(A)**, and between the model rats and benzbromarone-treated rats **(B)**. Differences are represented by the color of over-represented taxa: red indicating the allopurinol-treated rats or the benzbromarone-treated rats, green indicating the model rats. Circles represent phylogenetic levels from phylum (innermost circle) to genera (outermost circle).

After drug treatment, the gut microbiome was altered in the hyperuricaemia model rats. As shown in **Figure 4A**, allopurinol treatment caused the increase of Actinobacteria at the phylum level and its genera *Bifidobacterium* and *Collinsella*. Conversely, the phylum Lentisphaerae and five other genera (*Adlercreutzia*, *Anaerostipes*, *Bilophila*, *Desulfovibrio*, and *Morganella*) decreased due to allopurinol treatment. The significantly altered taxa with relative abundances above 1% were phylum Actinobacteria and genus *Bifidobacterium*, *Collinsella*, and *Desulfovibrio* (**Supplementary Figure S1**).

As shown in **Figure 4B**, the benzbromarone group exhibited increased abundance of the phyla Actinobacteria and Thermi and decreased abundance of Bacteroidetes and Lentisphaerae. At the genus level, *Bifidobacterium*, *Collinsella*, and *Proteus* levels increased after benzbromarone treatment, and *Adlercreutzia*, *Butyricimonas*, *Anaerostipes*, *Ruminococcus* levels decreased. The significantly altered taxa with relative abundances above 1% were phylum Actinobacteria and genus *Bifidobacterium*, *Collinsella*, and *Ruminococcus* (**Supplementary Figure S1**).

### Shared and Unique Altered Genera Among the Treatment Groups

**FIGURE 5:**
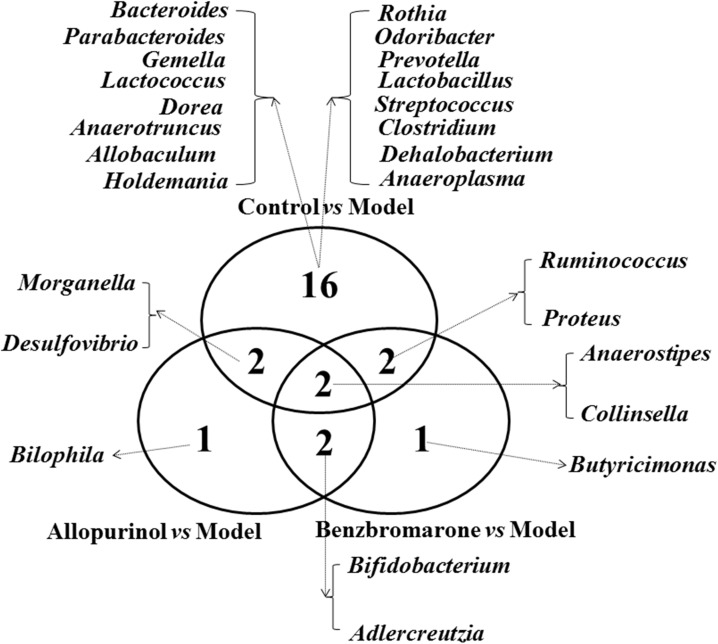
Venn diagram showing the distribution of shared and unique microbial genera in the control *vs* model, allopurinol *vs* model, and benzbromarone *vs* model comparisons.

To clearly show the alterations of gut microbiota associated with the treatment drugs, a Venn diagram was used to show the shared or unique altered genera of the gut microbiota. As shown in **Figure 5**, a total of 22, 7, and 7 altered genera were observed in the control *vs* model, allopurinol *vs* model, and benzbromarone *vs* model comparisons, respectively. Only two altered genera were shared by the three comparisons: *Anaerostipes* was more abundant and *Collinsella* was less abundant in the model group compared to the other three groups. Interestingly, both allopurinol and benzbromarone caused an increase of *Bifidobacterium* and a decrease of *Adlercreutzia*, but this alteration in genera was not observed in the control *vs* model pairing. Additionally, alterations in *Morganella* and *Desulfovibrio* were only shared by two comparisons (control *vs* model and allopurinol *vs* model), with both showing an increase in the model group. Alterations in two other genera (*Ruminococcus* and *Proteus*) were shared by two comparisons (control *vs* model and benzbromarone *vs* model), but the abundance of these genera were not consistent within the model group. The uniquely altered genera in the allopurinol *vs* model and benzbromarone *vs* model comparisons were *Bilophila* and *Butyricimonas*, respectively. Sixteen genera were uniquely altered in the control *vs* model comparison, including *Bacteroides*, *Lactococcus*, *Prevotella*, and *Clostridium*.

### Alterations of Metabolic Functions in the Gut Microbiota of Male Rats

**FIGURE 6:**
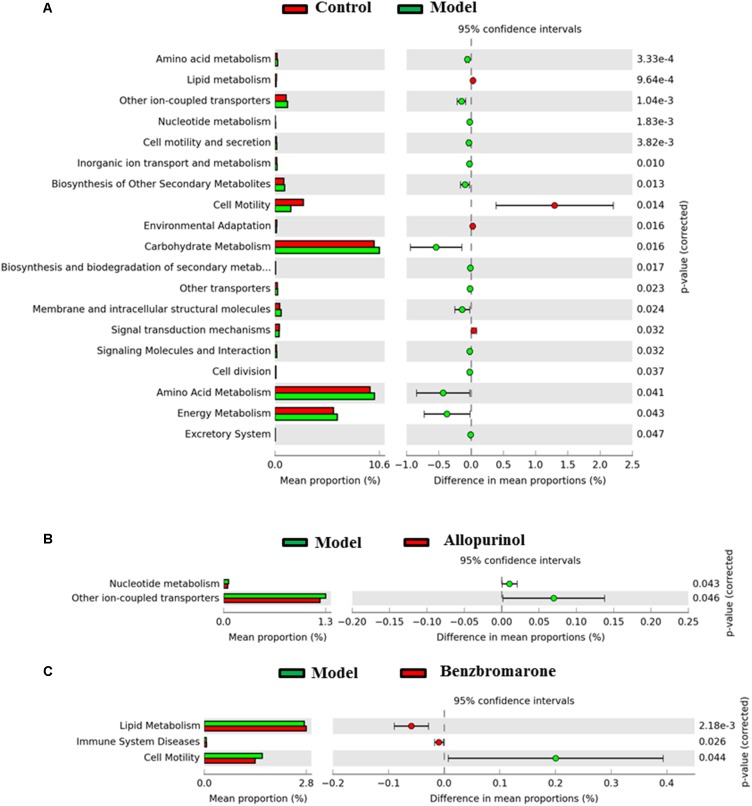
Predicted microbial functions comparisons. Gene functional categories were from level 2 of KEGG pathways. Gene functions with a significant difference are shown (adjusted *p* value <0.05). **(A)** Comparing microbial function between the control rats and model rats, the enriched functions in the control rats are shown with red color and the enriched functions in the model rats are shown with green color. **(B)** Comparing microbial function the model rats and allopurinol-treated rats, the enriched functions in the allopurinol-treated rats are shown with red color and the enriched functions in the model rats are shown with green color. **(C)** Comparing microbial function between the model rats and benzbromarone-treated rats, the enriched functions in the benzbromarone-treated rats are shown with red color and the enriched functions in the model rats are shown with green color.

Alterations in bacterial taxa also caused potential metabolic functions of the gut microbiota. As shown in **Figure 6A**, 19 KEGG pathways in level 2 were significantly distinct between the control and model groups. Increased KEGG pathways in the hyperuricaemia model rats were mainly related to carbohydrate, energy and amino acid metabolism, biosynthesis and biodegradation of secondary metabolites (**Figure 6A**). Additionally, the high-fat diets cause lipid metabolism, cell motility, environmental adaption and signal transduction mechanisms decreasing (**Figure 6A**).

After allopurinol treatment’ the gut microbiota exhibited significantly decreased abundance of nucleotide metabolism and ion-coupled transports pathways (**Figure 6B**). However, benzbromarone treatment caused the increases of lipid metabolism and immune system diseases pathways, and the decrease of cell motility pathways in the hyperuricaemia model rats (**Figure 6C**).

## Discussion

Recently, the gut microbiota has emerged as an important contributor to hyperuricaemia, and it has been shown to impact the response to disease treatment. Generally, the alterations of the gut microbiota observed after treatment may be attributable to alleviation of the disease (Halfvarson et al., 2017) or the effects of the treatment drugs (Wilson and Nicholson, 2017). Therefore, studying the association between gut microbiota and drug treatment could help to identify the biomarkers of disease remission or clarify the mechanism of the treatment drugs.

The differences in the gut microbiota between the control rats and the hyperuricaemia model rats were caused by diet; however, some alterations in the microbiota could also be related to hyperuricaemia. Several genera observed to decrease in the hyperuricaemia model function in purine absorption and UA decomposition, such as *Lactobacillus* (Yamada et al., 2016), *Streptococcus* (Mead, 1974), and *Clostridium* (Karlsson and Barker, 1949). Moreover, genera that increased in the hyperuricaemia model rats may be responsible for high UA levels. Bacteria of genus *Proteus* can convert purine into UA through secretion of xanthine dehydrogenase (Roxon et al., 1966). Several altered genera in the hyperuricaemia model rats are consistent with alterations of gut microbiome observed in gout patients (Guo et al., 2016). In addition, the gut microbiota is related to the disorders of carbohydrate, lipid, amino acid, and nucleotide metabolism, which were strongly associated with hyperuricaemia and UA (Frank, 1974; Marinello et al., 1978; Lima et al., 2015; Peng et al., 2015). In summary, the gut microbiota plays a role in elevating UA in the hyperuricaemia model rats.

In the hyperuricaemia model rats, allopurinol and benzbromarone were used to reduce UA. Both of these drugs caused alterations of the gut microbiota. Several alterations of the gut microbiota were shared by the allopurinol and benzbromarone treatment groups, including increased *Bifidobacterium* and decreased *Adlercreutzia Anaerostipes*. The above shared genera may be associated with the reduction of UA. The genus *Bifidobacterium* has been directly used as a probiotic therapy to alleviate hyperuricaemia (Cao et al., 2017). The genus *Anaerostipes* is known as a butyrate-producing bacterium (Bui et al., 2014), and butyrate may provide ATP energy for intestinal wall cells to excrete UA in the intestinal tract (Hosomi et al., 2012; Nieuwdorp et al., 2014). In addition, changes in these genera have previously been shown to be biomarkers of UA-related diseases. Chronic kidney disease patients were characterized by increasing levels of the genus *Adlercreutzia* (Xu et al., 2017). The alterations of the microbial genera shared by the allopurinol and benzbromarone treatment groups may be due to the alleviation of hyperuricaemia.

As two different drugs used to reduce UA, allopurinol and benzbromarone also impose different effects on the gut microbiota of hyperuricaemic rats. The unique alterations in genera induced by allopurinol or by benzbromarone should be due to the direct effects of the drugs. The genus *Bilophila*, the uniquely decreased genus in the allopurinol treatment group, is known to cause systemic inflammation (Feng et al., 2017). Benzbromarone treatment, conversely, uniquely caused a decrease of the genus *Butyricimonas*. Previous research has reported *Butyricimonas* as an infectious bacterium in the intestinal tract (Ogawa et al., 2018). Moreover, the alterations in the functions of the gut microbiota may explain different mechanisms of reducing UA between allopurinol and benzbromarone. Gut microbiota could convert nucleotide into UA and excrete UA to the outside of the bacterial cell through ion-coupled transporters (Krypotou et al., 2015). Therefore, the reductions of nucleotide metabolism and ion-coupled transporters caused by allopurinol may be beneficial for the reduction of UA in the intestinal tract. Benzbromarone renovated the disorders of lipid metabolism in the hyperuricaemia mode rats through the intervention of gut microbiota. Hence, the alterations in the gut microbiota are related to the different mechanisms of reducing UA between allopurinol and benzbromarone.

The gut microbiota plays roles in the induction of hyperuricaemia by high-fat diets and the reduction of UA by drugs. The components of the gut microbiota that are altered by high-diet feed are primarily involved in the disorders of purine, lipid, amino acid, and carbohydrate and energy metabolism in hyperuricaemia. Changes in genera after allopurinol and benzbromarone treatment are caused by the reduction of UA. Changes in genera that were unique to allopurinol or benzbromarone treatment may be attributed to the different mechanisms of reducing UA in male rats.

## Availability of Data and Material

The raw sequences of 23 male rats have been submitted to NCBI Project under accession number PRJNA479941 with NCBI Sequence Read Archive under accession number SRP152498.

## Ethics Statement

All animal handling and experimental procedures were performed in accordance with local ethical committees and the National Institutes of Health Guide for the Care and Use of Laboratory Animals. All efforts were made to minimize animal suffering and to reduce the number of animals used. All procedures performed in this study involving animals were approved by the Ethics Committee of Zhejiang Chinese Medical University.

## Author Contributions

ZH and CW conceived and designed the study, critically revised the manuscript, and were responsible for funding. YY and QL acquired and interpreted the data and drafted and critically revised the manuscript. HL critically revised the manuscript. All the authors read and approved the final manuscript.

## Conflict of Interest Statement

The authors declare that the research was conducted in the absence of any commercial or financial relationships that could be construed as a potential conflict of interest.

## Footnotes

1 http://huttenhower.sph.harvard.edu/lefse/
