# Supplementary material for: Alterations of the Gut Microbiome Associated With the Treatment of Hyperuricaemia in Male Rats
Source: Front Microbiol. 2018 Sep 19;9:2233. doi: 10.3389/fmicb.2018.02233 (PMC6156441; doi:10.3389/fmicb.2018.02233)
Supplement: Supplementary file 1 [file Data_Sheet_1.PDF]

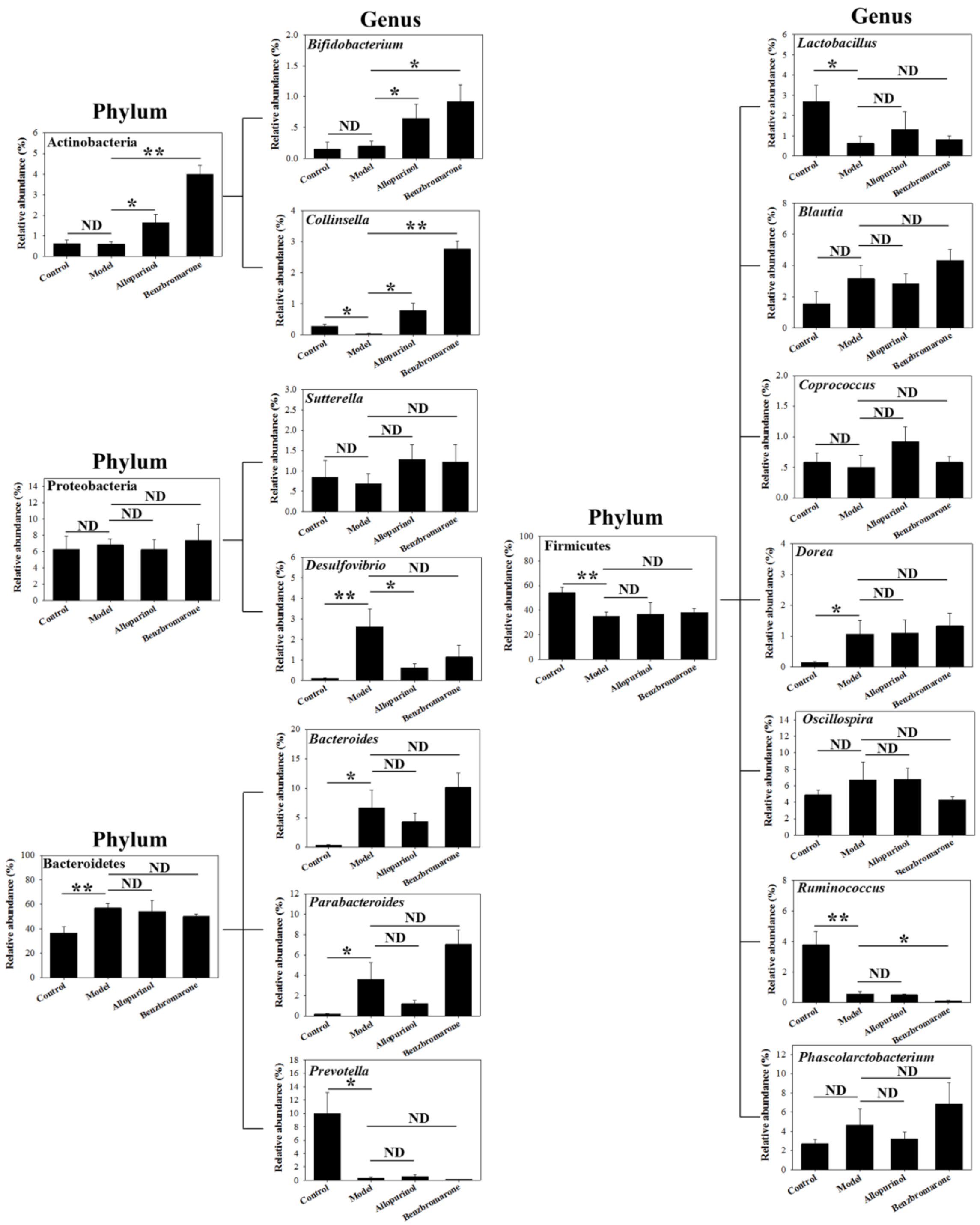

Figure S1 the taxa with relative abundances above 1% at the phylum and genus levels. “*” represents the adjusted *p* value <0.05 between two groups; “**” represents the adjusted *p* value <0.01 between two groups; “ND” represents the adjusted *p* value >0.05 between groups.
